# Comparison of Homo-Polyimide Films Derived from Two Isomeric Bis-Benzimidazole Diamines

**DOI:** 10.3390/molecules28134889

**Published:** 2023-06-21

**Authors:** Meng Lian, Feng Zheng, Lingbin Meng, Fei Zhao, Jun Liu, Jimei Song, Qinghua Lu

**Affiliations:** 1 Shandong Engineering Laboratory for Clean Utilization of Chemical Resources, Weifang University of Science and Technology, Weifang 262700, China; lianmeng@wfust.edu.cn (M.L.); mlb8124@126.com (L.M.); zhaofei@wfust.edu.cn (F.Z.); junliu@smail.nju.edu.cn (J.L.); songjimei1976@163.com (J.S.); 2 School of Chemical Science and Engineering, Tongji University, Siping Road 1239, Shanghai 200092, China; 3 Shanghai Key Laboratory of Electrical Insulation and Thermal Aging, School of Chemistry and Chemical Engineering, Shanghai Jiao Tong University, Shanghai 200240, China

**Keywords:** heteroaromatic polyimides, isomeric diamine, bis-benzimidazole

## Abstract

Heteroaromatic polyimides (PIs) containing benzimidazole have attracted tremendous attention due to their positive impact on the properties of PIs. Some research on PIs containing 4,4′-[5,5′-bi-1*H*-benzimidazole]-2,2′-diylbis-benzenamine (**4-AB**) has been reported. However, reports are lacking on homo-polyimides (homo-PIs) containing 3,3′-[5,5′-bi-1*H*-benzimidazole]-2,2′-diylbis-benzenamine (**3-AB**), which is one of the isomers of **4-AB**. In this paper, the influence of amino groups’ positions on the performance of homo-PIs was investigated. It was found that the net charge of the amine N group in **4-AB** was lower than that of **3-AB**, resulting in higher reactivity of **4-AB**. Consequently, PIs containing **4-AB** displayed better mechanical performance. Molecular simulation confirmed that **3-AB** and its corresponding PI chain exhibited distorted conformation, leading to the PI films containing **3-AB** having a lighter color. In addition, the **3-AB** structure was calculated to have higher rotational energy compared to **4-AB**, resulting in a higher glass transition temperature (*T*_g_) in PIs prepared from **3-AB**. On the other hand, PIs containing **4-AB** exhibited a higher level of molecular linearity, leading to a lower coefficient of thermal expansion (CTE) compared to PIs prepared from **3-AB**. Furthermore, all PIs showed higher thermal stability with a 5% weight loss temperature above 530 °C and *T*_g_ higher than 400 °C.

## 1. Introduction

Aromatic polyimides (PIs) are a family of high-performance polymers that are often synthesized through a two-stage polycondensation process involving a dianhydride and a diamine. These polymers exhibit excellent thermo-oxidative stability, superb mechanical properties, and unique electrical performance due to the strong intermolecular interactions originating from electronic polarization and intra- and interchain charge-transfer complex (CTC) formation [1]. Therefore, they have found widespread use in various industries, including electronics [2,3] and micro-electronics [4], aviation [5], gas separation [6], fuel cells [7], and many others. One method of endowing PIs with new functions or enhancing their original properties is through manipulation of their chemical structure. Heteroaromatic polyimides (PIs), which incorporate aromatic heterocyclic fragments such as benzimidazole [8,9], benzoxazole [10,11,12], pyridine [13], pyrimidine [14,15], and quinoxaline [16] into the PI backbones, have attracted tremendous attention because they combine the excellent properties of aromatic heterocyclic polymers with the easy processing of PIs.

**Figure 1:**
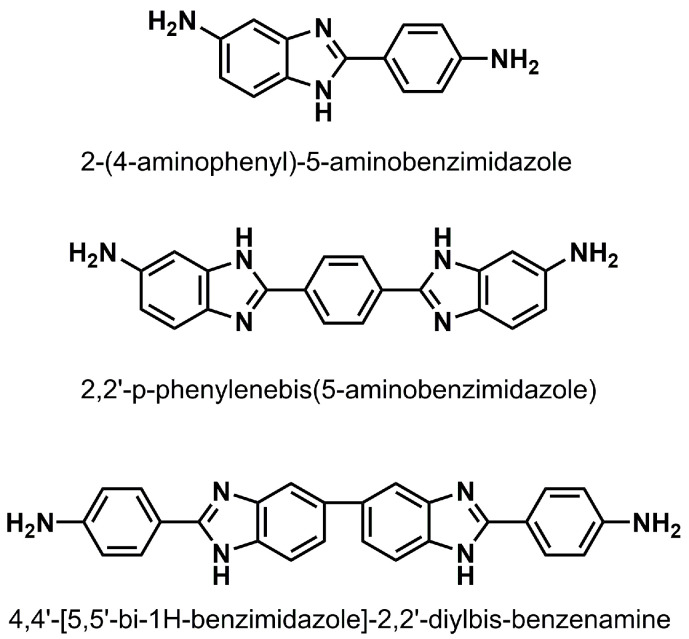
Chemical structure of diamine containing benzimidazole.

Considerable attention has been paid to heteroaromatic PIs due to their high molecular aromaticity and conjugation. Among them, heteroaromatic PIs containing benzimidazole have distinctive advantages, including the tendency to form hydrogen bonding interactions, and more facile synthesis methods. The most representative homo-polyimides (homo-PIs) [11,17] and co-polyimides (co-PIs) [18] are prepared from 2-(4-aminophenyl)-5-aminobenzimidazole (Figure 1). These PIs exhibit excellent thermal stability and a high modulus. In particular, benzimidazole moieties have been found to be the main contributing factor to their outstanding thermal and mechanical properties for PI system. In recent years, some bis-benzimidazole diamines were incorporated into the PIs backbone. Among them, PIs containing 2,2′-*p*-phenylenebis(5-aminobenzimidazole) (Figure 1) have been widely studied. The resulting homo-PIs [19] and co-PIs [20] showed significantly improved thermal and mechanical performance compared with those of common PIs and PIs containing benzimidazole, as expected. Another bis-benzimidazole diamine is 4,4′-[5,5′-bi-1*H*-benzimidazole]-2,2′-diylbis-benzenamine (Figure 1). Chen [21,22] and Ma [10] synthesized its corresponding homo-PIs and co-PIs, and reported that these PIs possess excellent thermal stability and mechanical properties due to enhanced molecular rigidity and macro-molecular interactions via hydrogen bonding. According to their structure–property relationships, the properties of the PIs are also affected by configuration change [20,23]. In our previous work [24], we investigated co-PIs containing the structural isomer of 4,4′-[5,5′-bi-1*H*-benzimidazole]-2,2′-diylbis-benzenamine, but there is still no research on the related homo-PIs.

In this work, the influence of amino groups’ positions on the properties of homo-PIs was investigated. To achieve this, 4,4′-[5,5′-bi-1*H*-benzimidazole]-2,2′-diylbis-benzenamine (**4-AB**) and its structural isomer 3,3′-[5,5′-bi-1*H*-benzimidazole]-2,2′-diylbis-benzenamine (**3-AB**) were synthesized using a one-step method. This method was found to be more facile and effective compared to the two-step procedure that involves the reduction of a nitro compound [19,22]. Two series of homo-PIs were synthesized, and molecular simulations were adopted to further determine the relationship between the monomer structure and the homo-PIs’ properties.

## 2. Results and Discussion

### 2.1. Monomer Characterization

**Figure 2:**
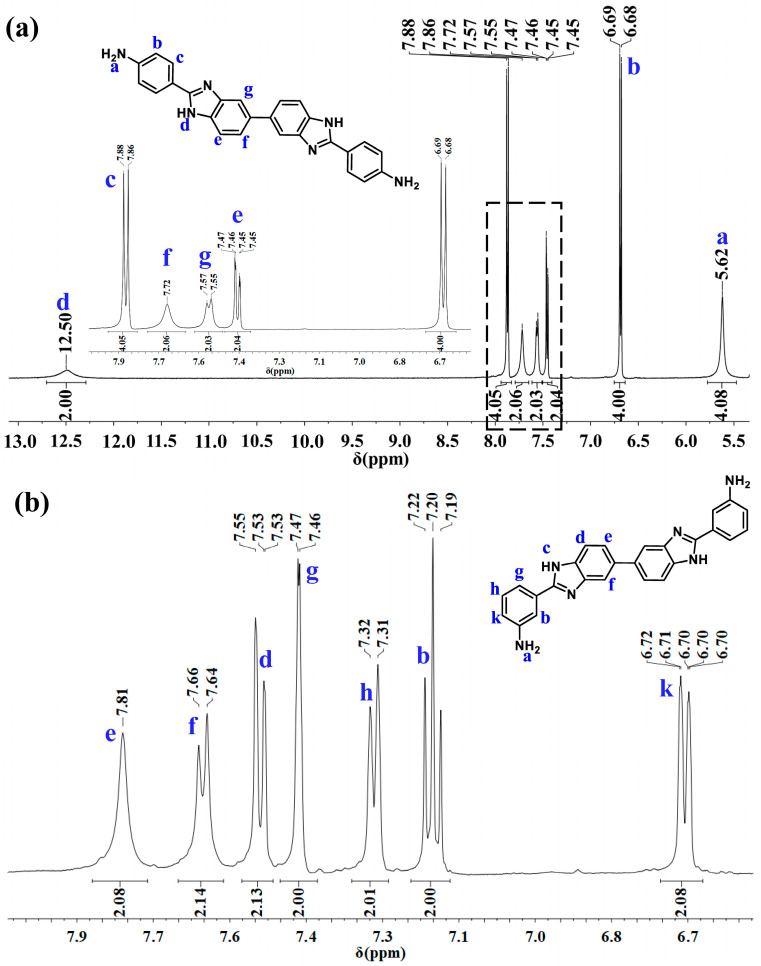
^1^H NMR spectrum of (**a**) **4-AB** (the inset figure was the curves of rectangular area range from 6.6 ppm to 8 ppm), and (**b**) **3-AB**.

**4-AB** and **3-AB** containing bis-benzimidazole moieties were designed and prepared from 3-aminobenzoic acid or 4-aminobenzoic via one-step dehydration condensation. The chemical structure of the synthesized monomer was characterized via ^1^H NMR, ^13^C NMR, and TOF-MS spectroscopy. The ^1^H NMR spectrum of **4-AB** and **3-AB** is given in Figure 2, while ^13^C NMR and TOF-MS spectroscopy are shown in Figures S1 and S2, respectively. The signals of aromatic protons in the phenyl structure appeared in the region of 8.00–6.50 ppm. Due to the intermolecular hydrogen bonds formed between -NH_2_ and N-H groups, the single broad peak resonating at 5.71 ppm was assigned to the protons in the amino groups, while the proton of N-H in benzimidazole was observed at 12.56 ppm. Moreover, the protons of amino and N-H groups in **3-AB** were not observed because the broad bands of the hydrogen bond and N-H with lower intensity were overlapped with the peaks of the aromatic protons. Finally, ^13^C NMR and TOF-MS confirmed that **4-AB** and **3-AB** were successfully synthesized.

### 2.2. Polymer Characterization

**Figure 3:**
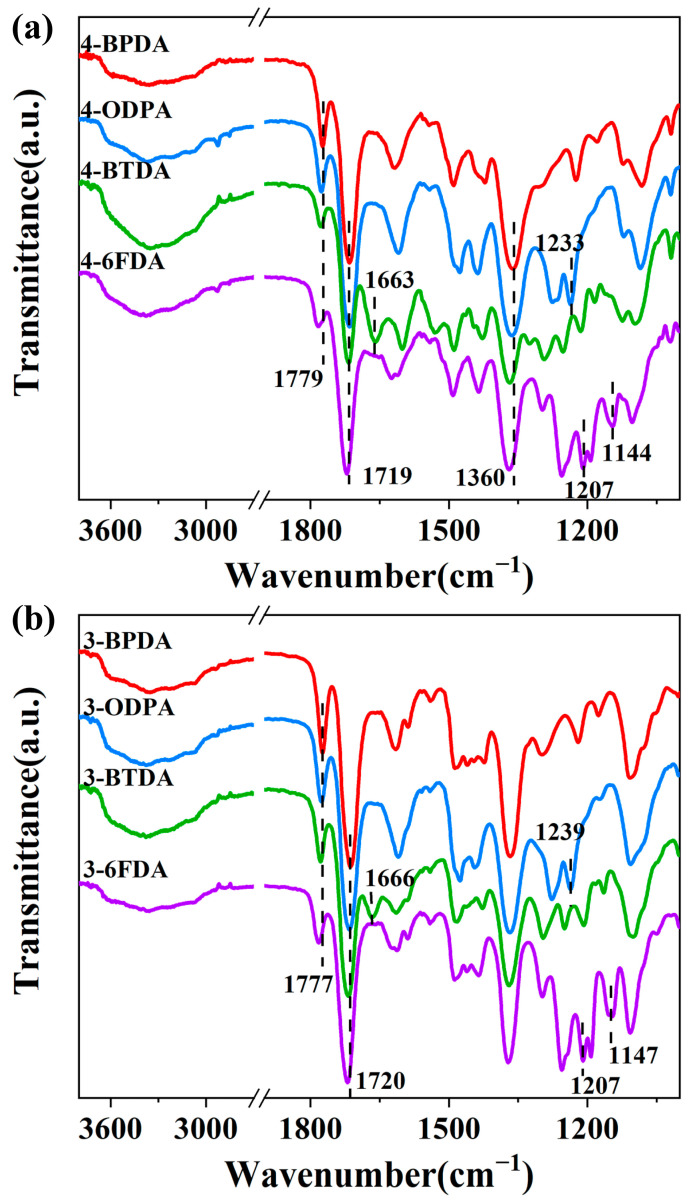
ATR-FTIR spectra of PIs containing (**a**) **4-AB** and (**b**) **3-AB**.

The anticipated chemical structures of the corresponding homo-PIs were confirmed via ATR-FTIR, as illustrated in Figure 3. All PIs exhibited a similar waveform, and the characteristic absorption of the PIs originated from the imide ring. The presence of peaks corresponding to C=O stretching bonds (~1779 cm^−1^ and ~1719 cm^−1^) and a C-N axial stretching bond (~1360 cm^−1^) in imide moieties indicated the formation of imide rings. With the exception of PIs prepared from dianhydride BTDA (i.e., 4-BTDA and 3-BTDA), there were no peaks observed around 1660 cm^−1^ and 1550 cm^−1^ for intermediate poly(amic acid), indicating that there was a nearly complete conversion of the poly(amic acid) precursor into PIs [25]. The obtained 4-BTDA and 3-BTDA also showed complete imidization, as evidenced by the presence of peaks at around 1663 cm^−1^ assigned to C=O in BTDA [26,27], while the peak at 1550 cm^−1^ for poly(amic acid) was hardly observed. The PIs of 4-ODPA and 3-ODPA showed typical C_arom_-O-C_arom_ vibration absorption signals at around 1233 cm^−1^ [28,29]. Additionally, for 4-6FDA and 3-6FDA, peaks at around 1207 cm^−1^ and 1144 cm^−1^ for-CF_3_ and C-F [30,31] vibration were also observed. All of these results suggest that the PIs were successfully prepared.

### 2.3. Film Quality

**Figure 4:**
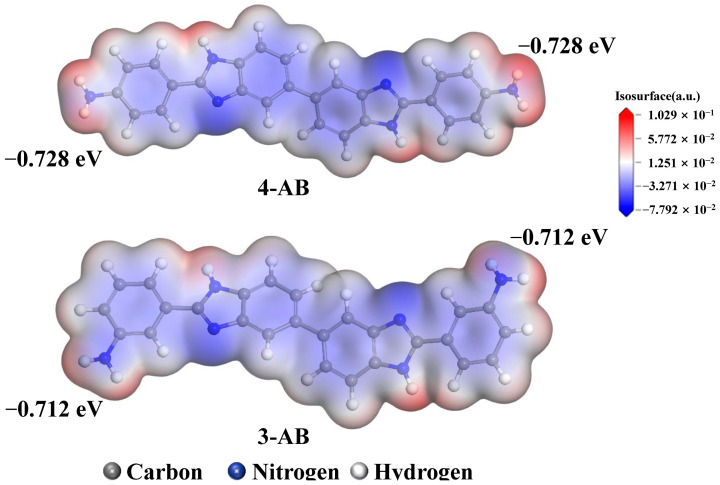
The net charges in the amine N atoms of **4-AB** and **3-AB**.

The molecular weight of a polymer is a crucial factor in ensuring the formation of polymer films. In this study, the molecular weight of poly(amic acid) is presented in Table 1, as PIs are insoluble and cannot undergo gel permeation chromatography analysis after thermal imidization. The number-average molecular weight (*M*_n_) of poly(amic acid) containing **4-AB** was in the range of 2.47 × 10^4^ g/mol to 3.21 × 10^4^ g/mol, and that of poly(amic acid) containing **3-AB** ranged from 1.97 × 10^4^ g/mol to 2.75 × 10^4^ g/mol. The formation of poly(amic acid) involved a process whereby the amino group carried out a nucleophilic attack on the carbonyl carbon. Differences in amine basicity, of which N atoms act as electron pair donors, can significantly affect the successful synthesis of poly(amic acid) and the molecular weight. Diamines with diverse configurations have different basicity, which affects the nucleophilicity and reactivity of **4-AB** and **3-AB**. The net charges on the amine N atoms are shown in Figure 4. The net charge of the amine N group in **4-AB** (−0.728 eV) was lower than in **3-AB** (−0.712 eV). Moreover, the lower net charge of the amine N group indicated a higher electron-donating ability. It can be speculated that the diamine **4-AB**, with the amino group in the *para*-position, possesses higher nucleophilicity than **3-AB**. As a result, the reactivity of the amino group in the *para*-position (**4-AB**) was higher than in the *meta*-position (**3-AB**), as evidenced by the molecular weight of poly(amic acid) containing **4-AB** being higher than that of poly(amic acid) containing **3-AB**.

**Figure 5:**
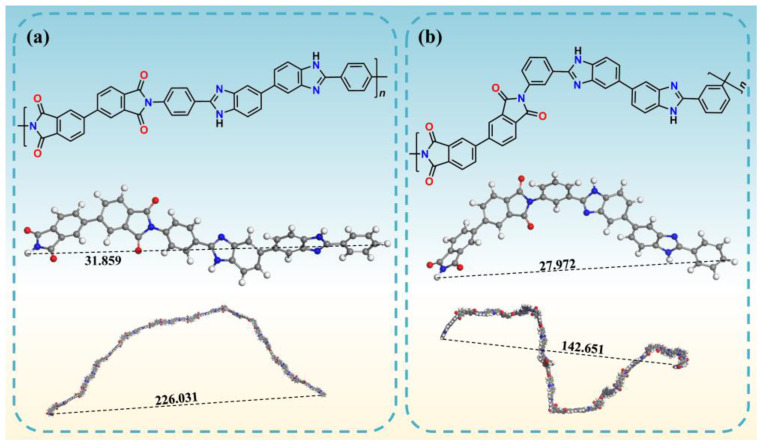
The chemical structure (**top**) and molecular simulation (**middle**) of repeating units, and single-chain simulation (**bottom**) of PI molecular chains: (**a**) 4-BPDA and (**b**) 3-BPDA.

The molecular weight of poly(amic acid) is sufficiently high to form self-standing polyimide films after undergoing thermal imidization. These films had a thickness ranging from 17 μm to 26 μm and were observed to have a dark-brownish or deep red color, which can be seen in 3.4 Polymer Synthesis part. Moreover, with the same dianhydride, the color of PIs containing **4-AB** was darker than that of PIs containing **3-AB**. Taking 4-BPDA and 3-BPDA as examples, their 3D chemical structures of one repeating unit, and simulated single-chain structures with 10 repeating units, are illustrated in Figure 5, and their corresponding simulated conformational parameters are listed in Table 2. The length of a 3-BPDA repeating unit was 27.972 Å, shorter than that of 4-BPDA (31.859 Å). The single-chain end-to-end distance of 3-BPDA was, in the same way, smaller than that of 4-BPDA. It can be inferred that the chain structure of PIs containing **3-AB** with amino groups in the *meta*-position exhibits a bent and distorted conformation. As is well known, the deep color of PIs is caused not only by their aromatic structure, but also by the formation of the intramolecular and intermolecular charge transfer complex (CTC). The larger conjugation system and charge transfer lead to a higher extinction coefficient, which was reflected in the intensive coloration of the PI films. The steric hindrance from the distorted **3-AB** could weaken the intramolecular and intermolecular CTC interactions. Therefore, the color of PIs containing **4-AB** was darker than that of PIs containing **3-AB** when polymerized with the same dianhydride.

Most of the PI films are tough and flexible, except for 4/3-6FDA. The 4/3-6FDA PIs form unbroken, self-standing thin films, but they become brittle when made into film rolls. The mechanical properties of the resulting PIs are listed in Table 3. The tensile strength, tensile modulus, and elongation at break were in the range of 83.2–117.4 MPa, 2.6–4.8 GPa, and 1.8–4.1%, respectively. From a structural point of view, the conformational rigidity of polymer chains was characterized by the Kuhn length [32,33], which is defined in Equation (1)

*b* = *h*^2^/*nl* (1)

where *b* is the Kuhn length, *h* is the end-to-end distance of the single chain, *l* is the length of one repeating unit, and *n* is the number of repeating units. In general, the Kuhn length of a polymer increases with the rigidity of its polymer chain. Table 2 shows that the Kuhn length of 4-BPDA is more than twice that of its structural isomer, 3-BPDA. This suggests that PIs containing 4-BPDA are more rigid than those containing 3-BPDA, when using the same dianhydride. The monomer **4-AB**, due to its more rigid and linear structure, and higher molecular weight, can provide more intermolecular CTC interactions. This leads to PIs with greater tensile strength and a slightly higher tensile modulus compared to **3-AB**, as can be seen in Table 3. Therefore, the amino groups at the *para*-position of the benzimidazole moieties are essential for improving the mechanical performances of PIs.

### 2.4. Thermal Properties

**Figure 6:**
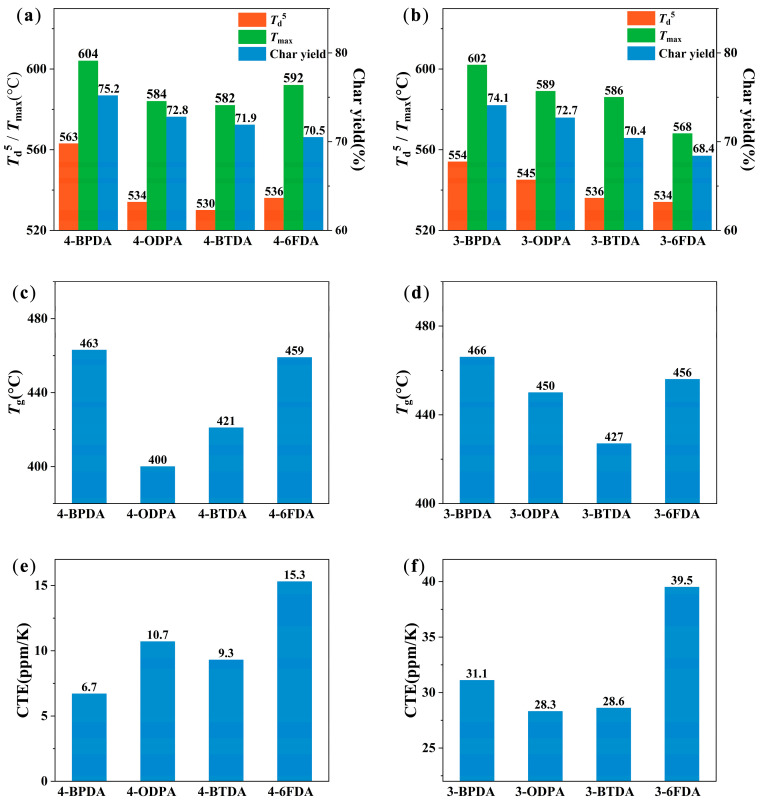
Thermal properties of PIs. Five percent decomposition temperature (*T*_d_^5^), temperature at which weight loss occurs at the maximum rate (*T*_max_), and char yield of PIs prepared from (**a**) **4-AB** and (**b**) **3-AB**. Glass temperature (*T*_g_) of PIs prepared from (**c**) **4-AB** and (**d**) **3-AB**. Coefficient of thermal expansion (CTE) of PIs prepared from (**e**) **4-AB** and (**f**) **3-AB**.

Thermal stability is a measure of the chemical resistance of a polymer to temperature. Usually, polymer chains may be cross-linked or degraded at high temperatures, leading to changes in their properties that can affect their applications [34]. Thermogravimetric analysis (TGA) is an effective method for studying the thermal stability of materials, and is generally measured by the temperature at which 5% weight loss occurs (*T*_d_^5^). The TGA curves were normalized to avoid moisture effects, as shown in Figure S3, and the related results are summarized in Figure 6a,b. The *T*_d_^5^ values of all PIs were above 530 °C, and the char yield at 800 °C ranged from 68.4% to 75.2%. Therefore, all PIs exhibited excellent thermal stability. Moreover, the *T*_d_^5^ values of 4-BPDA and 3-BPDA were higher than those of other PIs. This can be attributed to the higher bond energy of conjugated benzene rings in BPDA compared to non-ring-bridged structures [35]. The -C=O, -O-, and -CF_3_ structures were more likely to undergo molecular dissociation.

**Figure 7:**
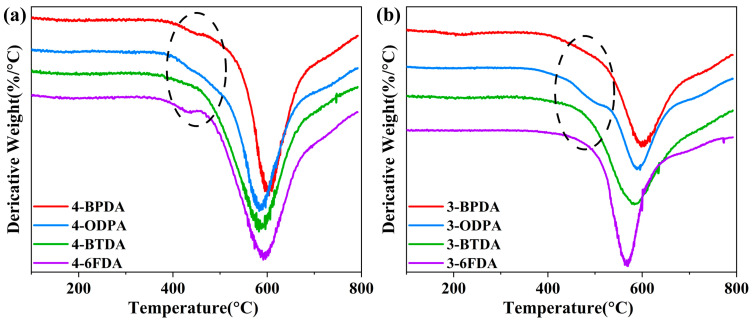
DTG curves of PI films containing (**a**) **4-AB** and (**b**) **3-AB** (The dashed elliptical area was the temperature between 400 and 500 °C which exhibited a slight weight-loss slope).

As is shown in Figure 6a,b, the temperature at which weight loss occurred at the maximum rate (*T*_max_) ranged from 568 °C to 604 °C. Although the TGA curves of the obtained PI films containing **3-AB** and **4-AB** were similar to those of traditional polyimides, their derivative thermogravimetric analysis (DTG) curves shown in Figure 7 indicate a slight weight-loss slope between 400 and 500 °C. This could be attributed to the decomposition of unstable N-H groups in the benzimidazole structure of these PIs. In a study on the thermal stability of polybenzimidazole polymers, it was found that the N-H groups in benzimidazole were prone to losing H atoms and dissociating at about 350 °C [36]. Therefore, the slight weight loss of PIs between 400 and 500 °C can be attributed to the free N-H dissociation. Furthermore, the N-H single bond, which has low bond energy, could form hydrogen bonds with C=O in the imide ring, thus enhancing the interaction between molecular chains and stabilizing the N-H structure. In 4-BTDA and 3-BTDA, not only could the imide ring provide C=O to form hydrogen bonds with N-H, but so could dianhydride BTDA. As a result, the first weight-loss process was not observed in 4-BTDA and 3-BTDA. There was also no obvious weight loss in 3-6FDA. This may be the result of N-H degradation accompanied by the degradation of other structures, such as CF_3_.

**Figure 8:**
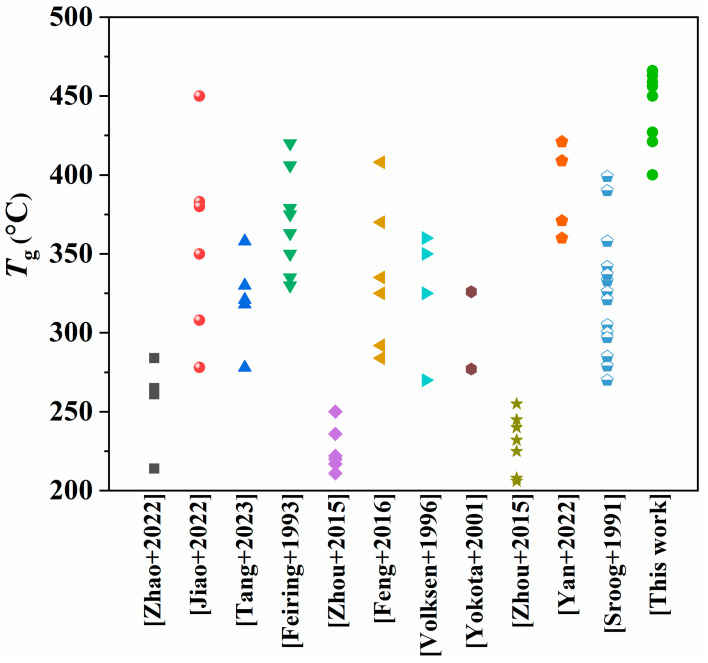
*T*_g_ contrast between our work and others published on homo-PIs [25,35,37,38,39,40,41,42,43,44,45].

The heat resistance of PI films can be characterized by their glass transition temperature (*T*_g_). Figure S4 illustrates the tan δ curves obtained by DMA for the PI films, where the change in the curve is attributed to the glass transition. Figure 6c,d demonstrate that the *T*_g_ values of all PIs were above 400 °C, with a range from 400 °C to 466 °C. This means they have high-level heat-resistance when compared to most reported homo-PIs, as shown in Figure 8 [25,35,37,38,39,40,41,42,43,44,45]. Notably, when the dianhydride is BPDA, the *T*_g_ of the corresponding PIs is the highest. For instance, the *T*_g_ of the PI containing **4-AB** was 463 °C, while the one containing **3-AB** reached 466 °C. On the other hand, in the PIs bearing **4-AB**, the lowest *T*_g_ (400 °C) was found in the one with ODPA dianhydride, while in PIs containing **3-AB**, the one with lowest *T*_g_ (427 °C) had BTDA dianhydride. The multi-ring structure of BPDA dianhydride is rigid, which means that the energy required for the movement of molecular chain segments is relatively high. Additionally, the conjugated BPDA structure is conducive to the formation of CTC interactions between molecular chains, leading to a higher *T*_g_ for the corresponding PIs. In contrast, dianhydride monomers containing flexible linkage groups (such as ether bonds, carbonyl groups, etc.) require less energy for the movement of the flexible structures, which can then drive the movement of the entire molecular chain segment. As a result, the *T*_g_ was slightly lower for these PIs containing ODPA, BTDA, and 6FDA.

**Figure 9:**
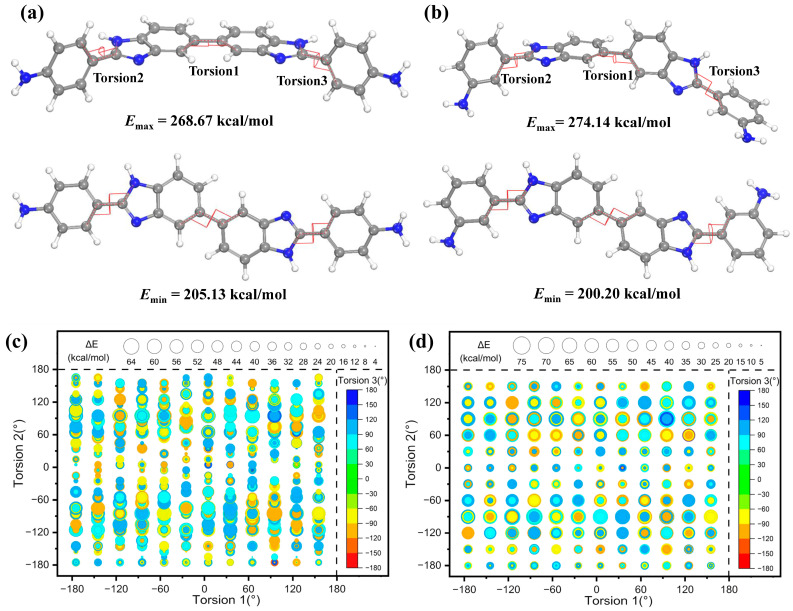
The structures of (**a**) **4-AB** and (**b**) **3-AB** with maximum (**upper**) and minimum (**lower**) rotational energy. Rotational energy changes in (**c**) **4-AB** and (**d**) **3-AB** when three torsions (red line: Torsion 1, Torsion 2, and Torsion 3) rotated simultaneously.

At the same time, although higher possible CTC formation in **4-AB** occurs than in isomer **3-AB**, the *T*_g_ of PIs films with isomeric diamine exhibited an obvious pattern: for PIs polymerized with the same dianhydride, the *T*_g_ of PIs derived from **3-AB** was slightly higher than that of PIs derived from **4-AB**. This difference can be attributed to variation in the rotational energy of molecular chain segments. From a molecular structure perspective, the glass transition temperature of a polymer indicates a relaxation phenomenon from an amorphous frozen state to a thawing state. When the polymer is gradually heated, the kinetic energy of its atoms and groups will increase until there is enough energy to drive the entire molecular chain segment and produce motion. Robert [46] notes that the rotation of the atoms and groups in polymer chain is essentially inhibited due to the insufficient rotational energy below *T*_g_. Therefore, the polymer with higher rotation energy is expected to have higher *T*_g_. Equation (2) was applied to express rotational energy [46]:

*H*_R_ = 0.5 *nRT*_g_ (2)

where *H*_R_ is the molar rotational energy, *R* is a constant, and *n* is the degree of freedom of the expressions of kinetic energy. Simulated calculations were performed to investigate the rotational energies of **4-AB** and **3-AB**. Considering that the rigid aromatic ring and heteroaromatic ring were difficult to rotate but vibrated, there were only three rotatable torsions occurring at the linking bond between benzene and benzimidazole rings, as shown in Figure 9a,b. The lowest rotational energy structure was designated as the reference point, set to zero, and the changes in rotational energy were recorded. The changes in rotational energy when the three torsions were rotated simultaneously are depicted in Figure 9c,d to illustrate the additional energy needed for rotation. The larger the diameter of the round bubble, the higher the rotational energy required. The maximum change in rotational energy for **3-AB** was 73.94 kcal/mol, which was higher compared to **4-AB** (63.54 kcal/mol). Additionally, the rotational energy changes in the three torsions rotating independently also demonstrated that the energy required for rotation in **3-AB** was higher than in **4-AB**, as shown in Figure S5. As a result, when polymerized with the same dianhydride, the PIs containing twisted **3-AB** required more energy for rotation, resulting in higher *T*_g_ values for the corresponding films.

The thermal expansion coefficient is an important parameter used to measure the dimensional stability of materials. In the fields of flexible copper-clad laminate (FCCL), solar panels, and display panels, the CTE of materials is the key parameter considered for structural design and process research. The lateral (in-plane) CTE curves of homo-PIs are shown in Figure S6, and the corresponding CTE values from 50 °C to 350 °C are shown in Figure 6e,f. The 4-BPDA PI displayed the best dimensional stability with the lowest CTE value (6.7 ppm/K), and the CTE value of 3-6FDA film was the highest at 39.5 ppm/K. Studies [47,48,49] have proven that CTE can be reduced when aromatic polyimides with rigid rod-like structures, linear polymer chains, and spontaneous in-plane orientation. As can be seen in Figure 5, the linearity of **4-AB** is higher than that of **3-AB**. Therefore, the CTE of PIs composed of **4-AB** is generally lower than that of PI films containing **3-AB** when polymerized with the same dianhydride.

## 3. Experimental Section

### 3.1. Materials

4-Aminobenzoic acid, 3-aminobenzoic acid, and 3,3′-diaminobenzidine were provided by J&K Scientific, and Poly(phosphoric acid) (PPA, 85 wt.%), anhydrous N,N-dimethylacetamide (**DMAc**), and dimethyl sulfoxide (**DMSO**) were provided by Adamas-beta^®^, Shanghai, China. Phosphorus pentoxide (P_2_O_5_) was acquired from Aladdin Biochemical Technology Co., Ltd., Shanghai, China. 3,3′,4,4′-Biphenyl tetracarboxylic dianhydride (**BPDA**), 3,3′,4,4′-benzophenonetetracarboxylic dianhydride (**BTDA**), 4,4′-oxydiphthalic anhydride (**ODPA**), and 4,4′-(hexafluoroisopropylidene)diphthalic dianhydride (**6FDA**) were obtained from ChinaTech (Tianjin) Chemical Co., Ltd., Tianjin, China.

### 3.2. Measurement

The structure of diamine monomers was characterized via ^1^H and ^13^C NMR spectra, which were recorded on a Bruker AVANCE III HD 500 system using DMSO-*d*_6_ as a solvent. TOF mass spectra were obtained using an ACQUI TYTM UPLC & Q-TOF MS Premier.

The structure of PIs was identified using a Nicolet 6700 infrared spectrometer in attenuated total reflectance mode (ATR-FTIR) over a range of 4000–650 cm^−1^ by accumulating 32 scans. The mechanical properties were collected using a WDW-50 universal electromechanical system at a strain rate of 5 mm/min. Dynamic mechanical analyses (DMA) were performed using Q800 at a heating rate of 5 °C/min and a load frequency of 1 Hz under a nitrogen stream. The decomposition behavior of PI films was measured using a Discovery TGA 550 with a temperature increase of 10 °C/min under nitrogen. Q400 TMA was applied to estimate the film’s in-plane coefficient of thermal expansion (CTE) at a heating rate of 5 °C/min, a 0.05 N static load, and a 50 mL/min nitrogen flow.

### 3.3. Monomer Synthesis

#### 3.3.1. Synthesis of 4,4′-[5,5′-Bi-1*H*-benzimidazole]-2,2′-diylbis-benzenamine (**4-AB**)

**Figure 10:**
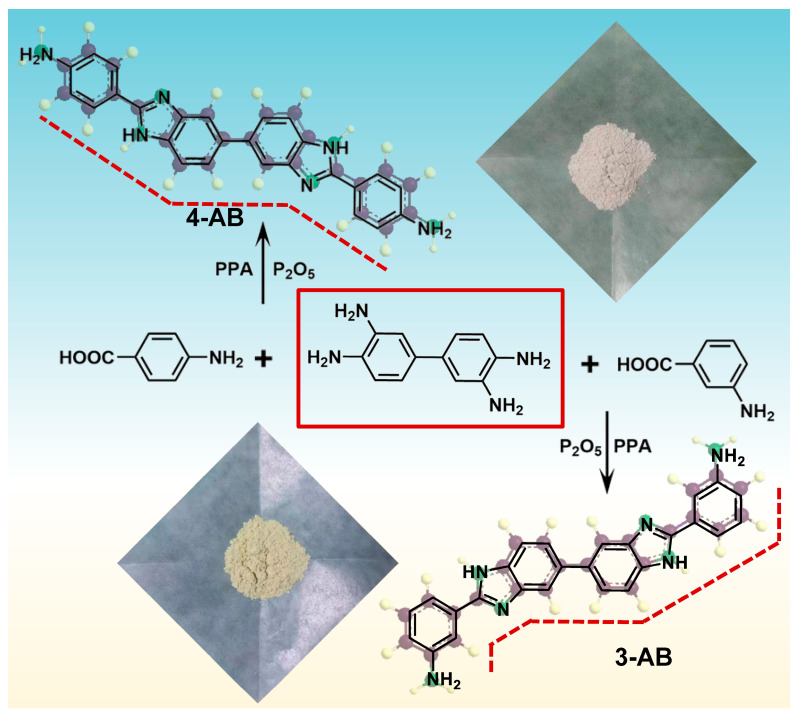
Synthesis and photograph of **4-AB** and **3-AB**.

Bis-benzimidazole diamine monomers were synthesized according to Figure 10. P_2_O_5_ (20 g) and PPA (160 g) were first added to a dried 500 mL three-neck flask equipped with a thermometer, a condenser, and a mechanical stirrer. The resulting mixture was heated to 100 °C and stirred to dissolve the P_2_O_5_ completely. 4-Aminobenzoic acid (13.7 g, 100 mmol) and 3,3′-diaminobenzidine (10.7 g, 50 mmol) were added into the mixture, and a thick paste was produced after stirring for 20 min at 100 °C. The mixture was allowed to react at 200 °C for 12 h. Then, it was cooled to 80 °C and poured slowly into ice-cold water. Finally, the precipitate formed and was neutralized to pH 8 using a 10% sodium hydrogen carbonate solution with rapid stirring. The crude product was recrystallized from ethyl alcohol to obtain a white solid (15.0 g, 72%), as shown in Figure 10. TOF-MS: *m*/*z* = 417.18; ^1^H NMR (500 MHz, DMSO-*d*_6_) δ 12.50 (s, 2H), 7.87 (d, *J* = 8.5 Hz, 4H), 7.72 (s, 2H), 7.56 (d, *J* = 7.6 Hz, 2H), 7.46 (dd, *J* = 8.3, 1.5 Hz, 2H), 6.69 (d, *J* = 8.6 Hz, 4H), 5.62 (s, 4H). ^13^C NMR (126 MHz, DMSO-*d*_6_): δ 158.37, 155.86, 140.45, 133.10, 132.96, 126.27, 122.49, 118.84.

#### 3.3.2. Synthesis of 3,3′-[5,5′-Bi-1*H*-benzimidazole]-2,2′-diylbis-benzenamine (**3-AB**)

Diamine **3-AB** was synthesized according to a procedure similar to that described for **4-AB**. The difference was in the use of 3-aminobenzoic acid instead of 4-aminobenzoic. As shown in Figure 10, a light-yellow solid (13.1 g, 63%) was obtained. TOF-MS: *m*/*z* = 417.18; ^1^H NMR (500 MHz, DMSO-*d*_6_) δ 7.81 (s, 2H), 7.65 (d, *J* = 8.2 Hz, 2H), 7.58–7.51 (m, 2H), 7.46 (d, *J* = 1.7 Hz, 2H), 7.32 (d, *J* = 7.5 Hz, 2H), 7.20 (t, *J* = 7.8 Hz, 2H), 6.76–6.67 (m, 2H). ^13^C NMR (126 MHz, DMSO-*d*_6_): δ 153.06, 149.54, 140.10, 139.28, 136.17, 130.97, 129.89, 122.26, 116.19, 115.83, 114.55, 113.13, 112.40.

### 3.4. Polymer Synthesis

**Figure 11:**
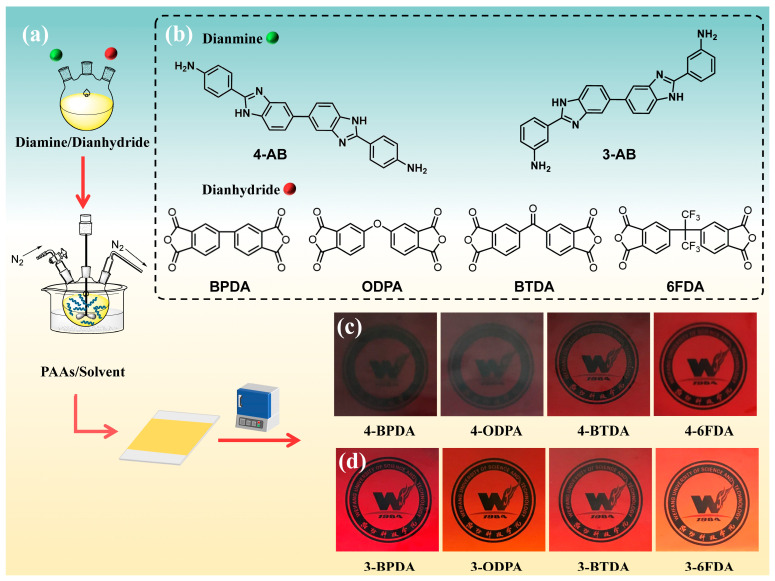
(**a**) The preparation technology of PIs. (**b**) Chemical structure of diamine and dianhydride. Visible images of PI films containing (**c**) **4-AB** and (**d**) **3-AB**. The Chinese characters in (**c**,**d**) represent the Chinese name of “Weifang University of Science and Technology”. These Chinese characters were seen through the PI films, and can be used as a reference for visual comparison of the darkness of the PIs films.

As shown in Figure 11a, the homo-PIs were synthesized via a conventional two-step polycondensation reaction. The diamine **4-AB** or **3-AB** with solvent (DMAc/DMSO = 2:1, *v/v*) was added into a three-necked flask with a mechanical stirrer. After the diamine was dissolved completely, several aromatic dianhydrides (BPDA, ODPA, BTDA, and 6FDA) were added and further stirred. The structures of diamine and dianhydride are shown in Figure 11b. After stirring at room temperature for 12 h, the poly(amic acid) solution was deformed and cast on clean glass substrates with a 400 μm depth blade. Then, the films were pre-heated and cured in a muffle with a program of 120 °C for 2 h, 150 °C for 2 h, 200 °C for 1 h, and 300 °C for 1 h. After cooling, the films were peeled by immersing them in hot water, and the corresponding images are shown in Figure 11c,d.

### 3.5. Molecular Simulations

The structures of diamine monomers were built using the Visualizer module of BIOVIC Materials Studio 2019. The net charges in the amine N atoms were calculated using the Dmol^3^ DFT program [50,51] via the GGA/PW91 function with a large basis set (DNP 4.4) [52]. Then, the monomers were used to build repeating units, which were connected using a homopolymer tool [53] to eventually establish long polyimide chains. The polymer chain in this work consisted of 10 repeating units according to the work on its predecessor [54,55].

## 4. Conclusions

A pair of structural isomers as diamine monomers (i.e., **4-AB** and **3-AB**) were prepared, and their corresponding homo-PIs were then synthesized with four dianhydrides. The experimental results showed that the reactivity of **4-AB** was higher than that of **3-AB**. Therefore, the tensile strength and tensile modulus of PIs prepared from **4-AB** were higher than those of **3-AB**. Due to their high molecular stiffness and conjugation, the *T*_d_^5^ of all the PIs was above 530 °C, and *T*_max_ was in the range of 568 °C to 604 °C. The DTG curves reflect that the N-H dissociation of bis-benzimidazole occured at 440 °C. Among these homo-PIs, the *T*_d_^5^ of 4-BPDA and 3-BPDA was higher than that of the other PIs. This is because the bond energy of conjugated benzene rings in BPDA is much higher than that of the flexible linkage structure in ODPA, BTDA, and 6FDA. The *T*_g_ of all PIs was higher than 400 °C; for example, the *T*_g_ of the 4-BPDA was 463 °C, and 3-BPDA reached 466 °C, achieving the highest level of heat-resistance of most reported homo-PIs. Furthermore, due to the higher molecular rotational energy of **3-AB**, the *T*_g_ of the corresponding PIs was higher than that of **4-AB**. The CTE of the PIs prepared from **4-AB** was lower than that of **3-AB** due to the linearity of **4-AB**.

## Figures and Tables

**Table 1:** Molecular weight of poly(amic acid) and film thickness.

	*M*_n_ ^a^ (×10^4^ g/mol)	*M*_w_ ^b^ (×10^4^ g/mol)	PDI ^c^	D ^d^ (μm)
4/3-BPDA	2.95/2.56	8.85/6.91	3.0/2.7	25/22
4/3-ODPA	2.78/1.97	9.45/7.09	3.4/3.6	26/20
4/3-BTDA	3.21/2.75	9.31/8.81	2.9/3.2	21/24
4/3-6FDA	2.47/2.32	8.65/7.89	3.5/3.4	19/17

^a^ *M*_n_: number-average molecular weight; ^b^ *M*_w_: weight-average molecular weight; ^c^ PDI: polydispersity index; ^d^ D: film thickness.

**Table 2:** Simulated conformational parameters.

	*l* ^a^ (Å)	*h* ^b^ (Å)	*b* ^c^ (Å)
4-BPDA	31.859	226.031	160.363
3-BPDA	27.972	142.651	72.749

^a^ *l*: repeating unit length; ^b^ *h*: distance between the ends of the chain; ^c^ *b*: Kuhn length.

**Table 3:** Film quality and mechanical properties of PIs.

	Film Quality	*σ* ^b^ (MPa)		*E* ^c^ (GPa)		*ε* ^d^ (%)	
		4-AB	3-AB	4-AB	3-AB	4-AB	3-AB
BPDA	F&T ^a^	117.4 ± 10.3	105.2 ± 11.3	4.8 ± 0.8	2.6 ± 0.4	2.6 ± 0.4	4.1 ± 0.3
ODPA	F&T	104.3 ± 12.6	91.9 ± 8.6	4.3 ± 0.6	3.9 ± 0.5	2.6 ± 0.5	2.3 ± 0.2
BTDA	F&T	91.2 ± 8.9	83.2 ± 7.9	3.5 ± 0.7	2.9 ± 0.5	2.5 ± 0.3	1.8 ± 0.4
6FDA	B&T	102.4 ± 7.6	98.5 ± 9.7	4.3 ± 0.9	3.1 ± 0.4	2.2 ± 0.4	3.1 ± 0.5

^a^ F: flexible, B: brittle, T: tough; ^b^ *σ*: tensile strength; ^c^ *E*: tensile modulus; ^d^ *ε*: elongation at break.

## Data Availability

The data presented in this study are available in this article and the Supporting Information.

## Supplementary Materials

The following supporting information can be downloaded at: https://www.mdpi.com/article/10.3390/molecules28134889/s1, Figure S1: The ^13^C NMR spectra of (a) **4-AB** and (a) **3-AB**; Figure S2: TOF-MS spectroscopy of (a) **4-AB** and (a) **3-AB**; Figure S3: TGA curves of PI films containing (a) **4-AB** and (b) **3-AB**; Figure S4: The tan δ curve f PI films containing (a) **4-AB** and (b) **3-AB**; Figure S5: (a) Rotational energy changes of three torsions in diamine when they rotate independently. The structure of (b) **4-AB** and (c) **3-AB** with corresponding torsions marked; Figure S6: In-plane TMA curve of PI films containing (a) **4-AB** and (b) **3-AB**.
