# Supplementary material for: Comparison of Homo-Polyimide Films Derived from Two Isomeric Bis-Benzimidazole Diamines
Source: Molecules. 2023 Jun 21;28(13):4889. doi: 10.3390/molecules28134889 (PMC10343788; doi:10.3390/molecules28134889)
Supplement: Supplementary file 1 [file molecules-28-04889-s001.zip › molecules-2445142-supplementary.pdf]

Supporting Information

Comparison of Homo-Polyimide Films Derived from Two Isomeric Bis-Benzimidazole Diamines

Meng Lian [1], Feng Zheng [2,*], Lingbin Meng [1], Fei Zhao [1], Jun Liu [1], Jimei Song [1] and Qinghua Lu [3,*]

[1] Shandong Engineering Laboratory for Clean Utilization of Chemical Resources, Weifang University of Science and Technology, Weifang 262700, China; lianmeng@wfust.edu.cn (M.L.); mlb8124@126.com (L.M.); zhaofei@wfust.edu.cn (F.Z.); junliu@smail.nju.edu.cn (J.L.); songjimei1976@163.com (J.S.)
[2] School of Chemical Science and Engineering, Tongji University, Siping Road 1239, Shanghai 200092, China
[3] Shanghai Key Laboratory of Electrical Insulation and Thermal Aging, School of Chemistry and Chemical Engineering, Shanghai Jiao Tong University, Shanghai 200240, China
* Correspondence: fzheng@tongji.edu.cn (F.Z.); qhlu@sjtu.edu.cn (Q.L.)

Content

Figure S1. The ^{13}C NMR spectrum of (a) **4-AB** and (a) **3-AB**.

Figure S2. TOF-MS spectroscopy of (a) **4-AB** and (a) **3-AB**.

Figure S3. TGA curves of PI films containing (a) **4-AB** and (b) **3-AB**.

Figure S4. The tan δ curve f PI films containing (a) **4-AB** and (b) **3-AB**.

Figure S5. (a) Rotational energy changes of three torsions in diamine when they rotate independently. The structure of (b) **4-AB** and (c) **3-AB** with corresponding torsion marked.

Figure S6. The in-plane TMA curve of PI films containing (a) **4-AB** and (b) **3-AB**.

^{13}C NMR was used to further confirm the structure of **4-AB** and **3-AB**. In organic compounds containing nitrogen, because the relaxation time of individual carbon atoms in the molecule is too long, the peaks of carbons near nitrogen were always wide and low, or even disappear. Therefore, in Figure S1(a), the number of carbon peaks was less than that of the **4-AB** carbon atom. The number of carbon peaks in **3-AB** was consistent with the structure of **3-AB**, whereas some peaks were broad and low in Figure S1(b). On the other hand, the peak of carbon bonded to the nitrogen atoms usually appeared at a low field. Therefore, the peak at 158.37 ppm for **4-AB** and 153.06 ppm for **3-AB** was assigned to the carbon a in the imidazole ring. The peak at 155.86 ppm and 149.54 ppm were assigned to the carbon b bonded the amine group. Moreover, the signals of carbon atoms in the phenyl structure appeared in the region of 110-135 ppm.

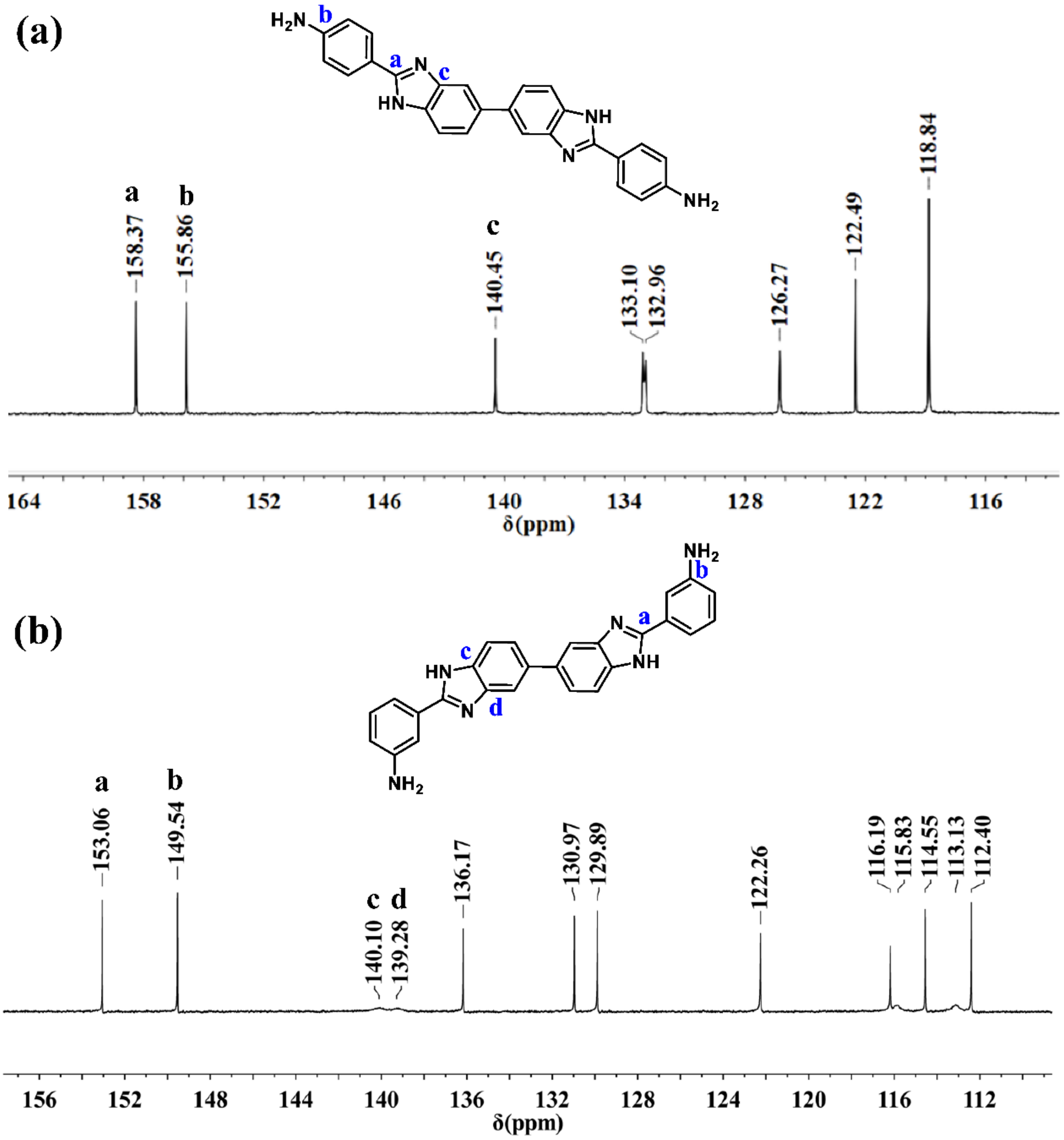

Figure S1. The ^{13}C NMR spectrum of (a) **4-AB** and (b) **3-AB**.

TOF mass spectra were also applied to confirm the structure of **4-AB** and **3-AB**. The peak of *m*/*z* at 417 was assigned to the [M+H], while *m*/*z* at 209 was assigned to [1/2M + H]. Usually, in compounds with higher conjugation system, or contained the active hydrogen atoms from amide, carboxyl, hydroxyl group, *etc*. the peak of 2M may appear. For **4/3-AB**, the structure exhibits high conjugation, and N-H in benzimidazole and amine group could provide active hydrogen bond. Therefore, the peak of m/z at 833 was assigned to [2M + H].

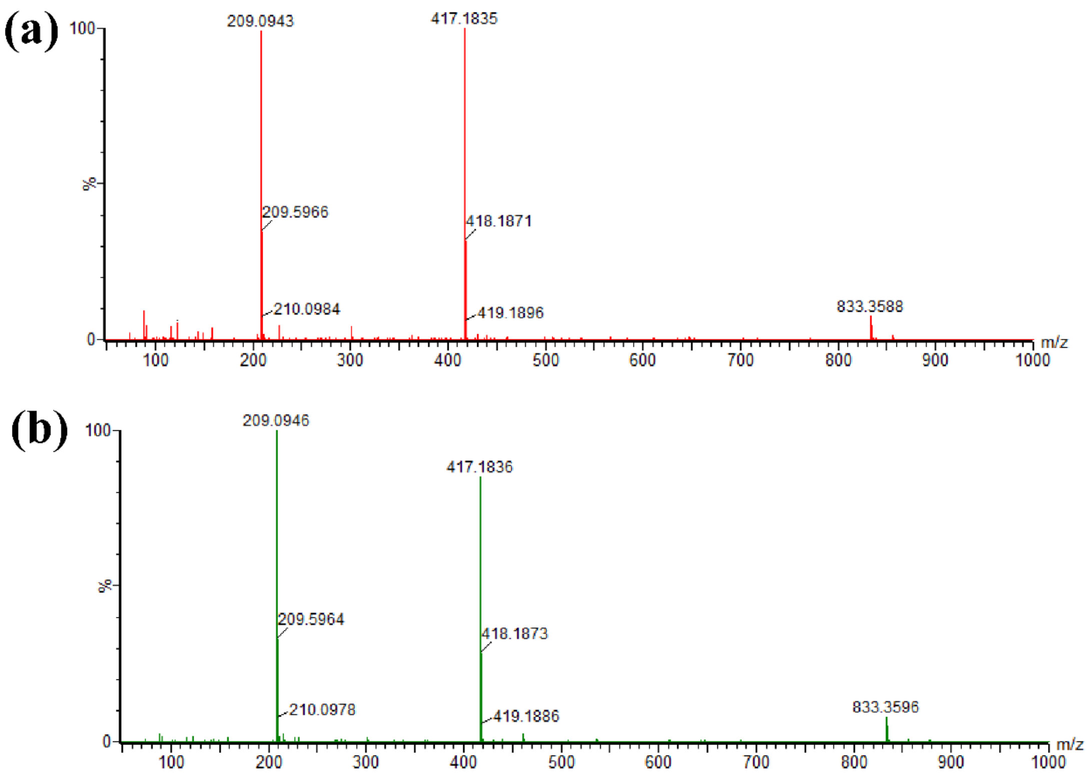

Figure S2. TOF-MS spectroscopy of (a) **4-AB** and (a) **3-AB**.

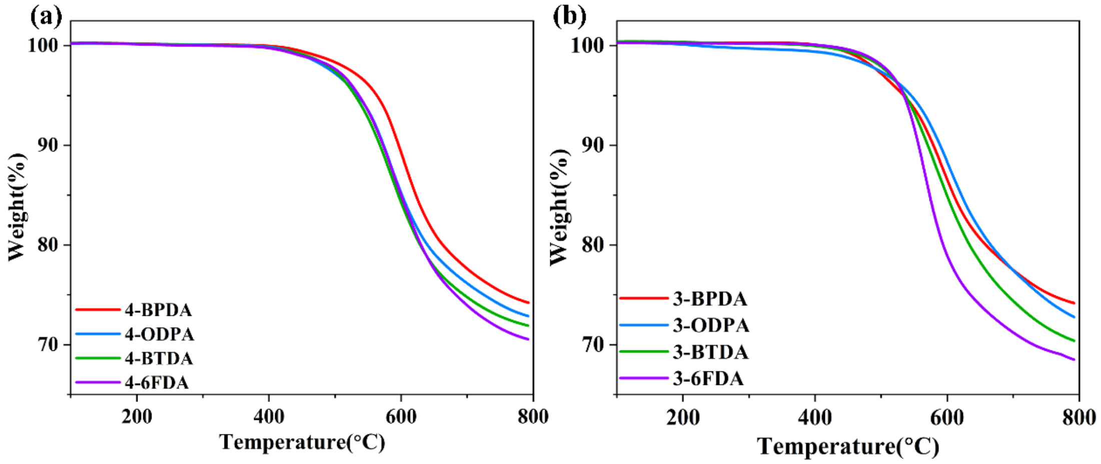

Figure S3. TGA curves of PI films containing (a) **4-AB** and (b) **3-AB**.

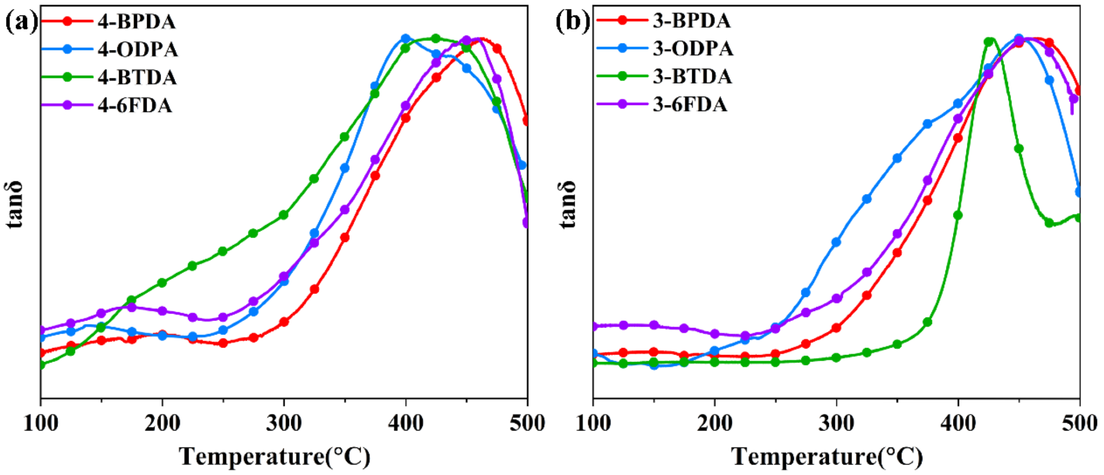

Figure S4. The tan δ curve f PI films containing (a) **4-AB** and (b) **3-AB**.

As shown in Figure S5. due to the symmetrical structure of **4-AB** and **3-AB**, the curve of Torsion 2 and Torsion 3 was similar, and the energy needed for rotation was higher than Torsion 1. The highest energy for **3-AB** was from **3-AB-2** (Torsion 2), higher than that of **4-AB** which was from **4-AB-2** (Torsion 2) or **4-AB-3** (Torsion 3). Therefore, the rotational energy changes of the three torsions rotating independently also demonstrated that the energy required for rotation in **3-AB** was higher than in **4-AB**.

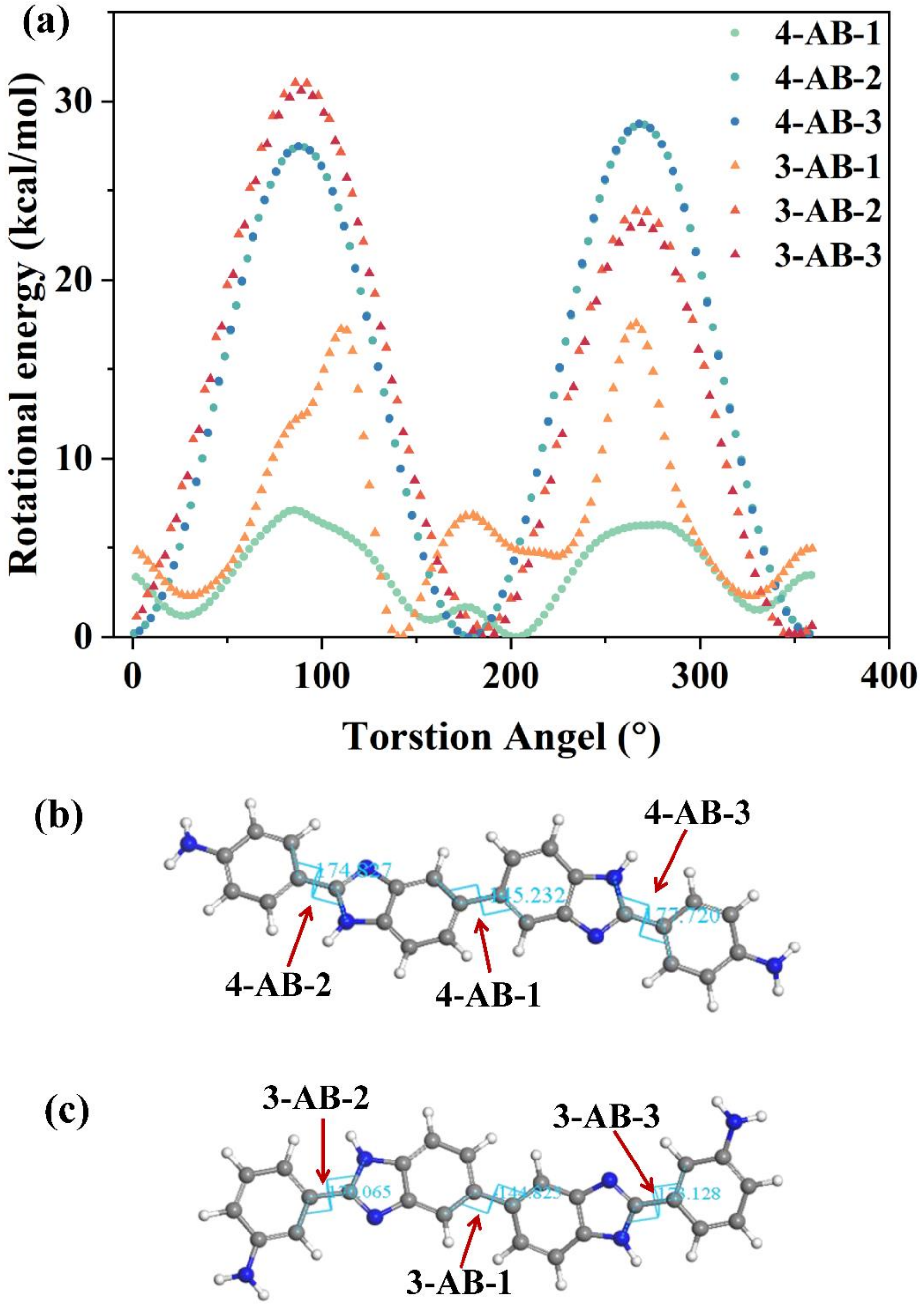

Figure S5. (a) Rotational energy changes of three torsions in diamine when they rotate independently. The structure of (b) **4-AB** and (c) **3-AB** with corresponding torsion marked.

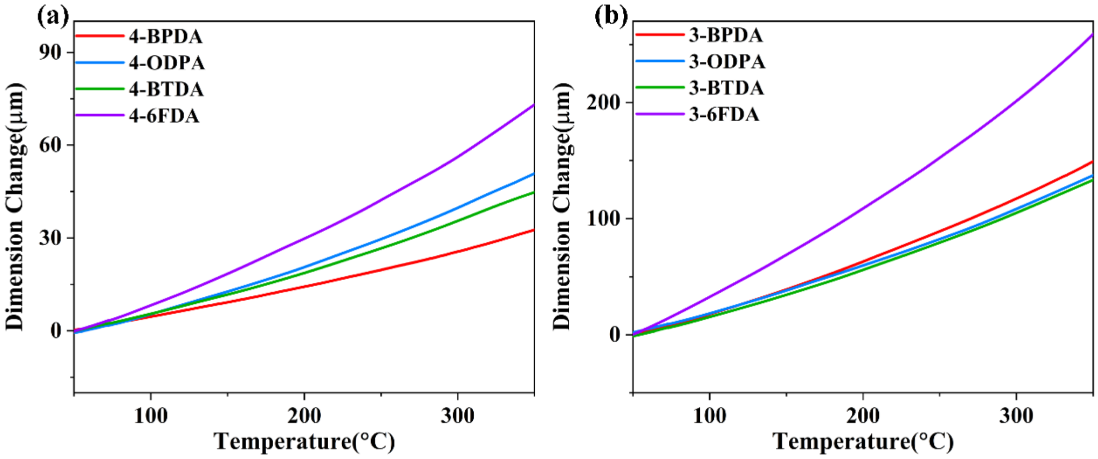

Figure S6. The in-plane TMA curve of PI films containing (a) **4-AB** and (b) **3-AB**.
